# From Urban village housing to tenant mental health: the crucial role of community attachment in Chinese megacities

**DOI:** 10.3389/fpubh.2025.1490105

**Published:** 2025-03-06

**Authors:** Junrui He, Hongsheng Chen, Yidan Cai, Zheng Li, Jianping Ye

**Affiliations:** ^1^School of Public Administration and Policy, Renmin University of China, Beijing, China; ^2^School of Architecture and Urban Planning, Shenzhen University, Shenzhen, China; ^3^School of Architecture and Urban Planning, Shenzhen University, Shenzhen, China

**Keywords:** Urban villages, housing quality, community attachment, tenant mental health, megacities, Shenzhen

## Abstract

Housing has been a longstanding social issue in China’s megacities, profoundly affecting the residents’ quality of life. Urban villages in megacities, despite their substandard living conditions, provide affordable housing for many residents. The study of living conditions and their social implications in urban villages has been a central theme in Chinese urban research. However, previous research on Chinese urban villages has paid less attention to the relationship between tenants’ mental health, housing quality and community attachment. This study selects seven typical urban villages in Shenzhen as the study cases, collecting first-hand anonymous questionnaire data to study tenants’ mental health status and explore the mediating role of community attachment in the relationship between housing quality and residents’ mental health. The finds show that the housing quality ( β = 0.228, *p* = <0.05) and housing affordability ( β = 0.196, *p* = <0.05) have a significant effect on tenants’ mental health. Specifically, better the housing quality and lower the housing affordability are associated with improved mental well-being among urban village tenants. Additionally, community attachment ( β = 0.416, *p* = <0.05) has a significant positive impact on tenants’ mental health, and serves as an important mediating factor in the relationship between housing quality and tenants’ mental health. This study proposes that improving living quality in megacity urban villages and creating a favorable living environment can increase tenants’ attachment to urban villages, and significantly improve their mental health. These factors should be emphasized in the current urban renewal policy for Chinese megacities.

## 1 Background

China’s rapid economic development over the past few decades has driven an substantial migration from rural areas and small towns to large, economically prosperous cities in pursuit of employment opportunities and a better quality of life. According to China’s seventh population census (2020), the urbanization rate has exceeded 60%. The migrant population is an important force driving the rapid development of cities, but due to the concentrated flow of migration in China, the housing supply in megacities has long been insufficient. As a result, housing shortages remain one of the most significant barriers to the integration of China’s so-called “migrant population” into megacities (1). Faced with unaffordable housing prices in megacities, many urban dwellers opt for rental accommodation. Urban villages are a special type of settlement in the structure of China’s megacity housing market. Unlike commercial residential property, urban villages generally offer substandard living conditions and lack essential public services, leading to a high concentration of cheap rental housing (2). As urban villages accommodate a large number of low-and middle-income earners and “urban outsiders,” they have become a long-standing social issue in China (3).

Urban villages are a unique spatial phenomenon emerging from China’s urbanization process (4). Due to the dualistic structure of the land system (division between collective and state-owned land), some villages that have not been expropriated and demolished by the government have gradually been surrounded by urbanized areas. As a result, urban villages are often surrounded by a large number of high-rise buildings, and many are located next to the urban central district (5). Their close proximity to major employment centers, coupled with affordable rental costs, renders them particularly appealing to low-income groups (6). However, living conditions in urban villages are often substandard, with common issues including overcrowding, environmental pollution, high building density and inadequate infrastructure.

While numerous studies have focused on the living conditions of urban village residents (2), few scholars have paid attention to the community attachment and mental health of residents. Despite poor living conditions, urban villages often function as long-term residences for many migrants, effectively becoming an alternative home. As a result, the extent of tenants’ community attachment to urban villages has remained a subject of debate in the academic literature (7, 8). Therefore, it is necessary to consider the role of community attachment in shaping the relationship between housing quality and mental health of tenants, to gain a deeper understanding of the real living conditions of urban village tenants in megacities.

This study investigates the relationship between housing quality in urban villages and tenants’ mental health, with a particular focus on the mediating role of community attachment. A survey was conducted in seven typical urban villages in Shenzhen, one of China’s most densely populated megacities. Despite its economic prosperity, Shenzhen faces urban challenges, such as high property prices, housing affordability issues. Against this background, Shenzhen’s urban villages have played an important role in housing supply providing shelter to a large migrant population (9, 10). According to the *Shenzhen Urban Village Building Dictionary 2022*, issued by the China Development Institute (Shenzhen), Shenzhen’s urban villages accounts for 36.3% of the city’s total housing floor area, and may be able to accommodate approximately 10 million residents. Recognizing their role in relieving housing pressures, the Shenzhen government has implemented various renewal policies to promote the housing quality. In recent years, Shenzhen has promoted ‘the unified renovation and unified leasing’ model for urban villages, allowing private enterprises to participate in housing improvement. While this policy has enhanced housing conditions, it has also led to rental costs. Given the large number of long-term residents in urban villages, Shenzhen provides an ideal case for studying the impact of the living environment on tenants’ mental health. The findings of this study can also provide urban renewal policies for urban villages in China.

## 2 Literature review and research hypotheses

### 2.1 The relationship between housing quality and residents’ mental health

China’s rapid urbanization over the past few decades has driven large-scale migration from the countryside to the cities, creating a unique landscape of urban villages under the dualistic land system (11). In particular, the growing population size of China’s megacities have resulted in migration into these economically prosperous cities, exacerbating the tension between population expansion and housing supply. Due to limited affordable housing resources in megacities, urban villages with substandard living conditions provide a large amount of cheap housing for the migrant population (3).

However, these cheap housing units in urban villages are often widely criticized for their cramped living space, poor community living environment, lack of public services, low-quality construction, safety concerns etc. (12, 13). Many scholars have conducted extensive research on the housing quality in urban villages. For example, Li et al.’s study in Xiamen showed that the poor indoor environment of low-cost housing in urban villages had significant negative health effects on tenants (14). However, some researchers have suggested that many migrant workers are more concerned about the distance from their place of employment and the cost of living than the housing quality (15). Without urban villages, many low-income migrant workers would be unable to sustain their livelihoods in the city (*Ibid*).

Therefore, the first question is whether the housing quality in urban villages has an impact on the mental health of the residents, a topic that remains controversial in the literature. While poorer housing quality can affect the mental health of residents in some urban villages to a certain extent (32), housing conditions vary significantly across different cities, and their effects on tenants may differ. In particular, in Chinese mega-cities such as Shenzhen, urban villages have long served as key settlement for migrants, playing a crucial role in facilitating the integration of low-income groups into the urban fabric. Therefore, we first test the relationship between objective living conditions and tenant mental health in megacity urban villages.

Based on established research, this study proposes hypothesis 1: *Better housing quality in urban villages can significantly and positively affect tenants’ mental health (H1).*

While the literature on housing quality provides valuable insights into how the physical characteristics of living environments directly influence mental health outcomes, it is important to recognize that residents’ well-being is also shaped by the economic burden of housing. In the context of urban villages—where many tenants face financial constraints—the affordability of housing emerges as a critical factor that may compound or mitigate the effects of substandard living conditions. The next section, therefore, shifts the focus from the physical attributes of housing to examine how housing affordability influences residents’ mental health.

### 2.2 The relationship between Housing affordability and residents’ mental health

The large number of jobs in megacities serves as a major driver of sustained population inflows. However, a number of studies have shown that urban residents have a higher risk of mental illness than rural residents (16). Several scholars have conducted extensive research on the relationship between community living environments and residents’ mental health. For example, Ma et al. (2018) examined the association between various types of noise pollution and mental health symptoms in Beijing residents, finding that higher perceived exposure to noise pollution was significantly correlated with poorer mental health (33).

In addition, housing conditions, which are related to the costs and quality of life of urban residents, have also been cited by many scholars as an influencing factor on the mental health of residents. As Seo and Park (17), in their study of the Survey of Living Conditions and Welfare Needs of Korean Adolescents (*n* = 1,308), found that material hardship caused by housing cost burdens was negatively associated with mental health in single-person households (17). Xiao et al. (2018) used a structural equation modeling approach to explore the impact of housing conditions on mental health among Shanghai’s migrant population, demonstrating that housing conditions have a direct impact on mental health. Their findings indicated that housing conditions have an indirect impact on mental health through neighborhood satisfaction (34).

While many migrants in China’s megacities reside in urban villages—whose substandard housing conditions have been widely criticized—their affordability remains a crucial yet often overlooked factor. Therefore, it is necessary to consider housing affordability as an influencing factor, while many migrants in China’s megacities reside in urban villages—whose substandard housing conditions have been widely criticized—their affordability remains a crucial yet often overlooked factor.

Accordingly, this paper proposes hypothesis 2: *Housing affordability is negatively correlated with tenant mental health in megacity urban villages (H2)*.

Although housing affordability is a key determinant of mental health—reflecting the financial pressures faced by residents—it does not capture the full spectrum of psychosocial factors inherent in urban living. In urban villages, the emotional bonds and sense of belonging that tenants develop with their communities can also have a profound impact on their mental well-being. Consequently, the subsequent section explores the role of community attachment, aiming to elucidate how these emotional ties may mediate and moderate the relationship between housing conditions and mental health outcomes.

### 2.3 The role of community attachment in urban villages

Community attachment is an important dimension in evaluating the relationship between residents and their community. As the predecessor of the urban village was a traditional rural community with a clan-based society, indigenous residents maintain strong emotional bonds and economic ties to their communities. However, for the large number of non-local tenants renting in urban villages, their emotional connections to urban villages are complicated (18).

On the one hand, for many low-income earners, urban villages serve as long-term places of residence, where their living conditions are closely related to the surrounding environment. On the other hand, within a specific institutional context, they are often regarded as “outsiders” or “strangers to the city” and may leave the urban villages at any time, either voluntarily or involuntarily (19, 20). In this scenario, the nature of tenants’ community attachment to urban villages remains a subject worthy of further exploration.

Du and Li’s (21) study on migrants in Guangzhou’s urban villages found that these settlements provide a transitional refuge for migrants’ integration into urban life. Their findings further indicate that community qualities and community relations influence migrants’ sense of attachment to urban villages. Similarly, Chang et al. (22) demonstrated that housing conditions, neighborhood environments, and social relationships are positively related to community attachment, with variations in social relationships and physical environments leading to differing level of community attachment. Liu et al. (23) concluded that residential uncertainty and poor neighborhood environments reduce migrants’ sense of urban belonging.

Given that community attachment has a positive impact on residents’ life status, it is also shaped by external living conditions and, in turn, may affect residents’ mental health. Accordingly, we used community attachment as a mediating and moderating variable to analyze its role in the relationship between housing quality and tenants’ mental health in urban villages.

Thus, the third hypothesis proposed in this study is: *Community attachment mediates and moderates the relationship between housing quality and tenant mental health (H3)*.

**Figure 1:**
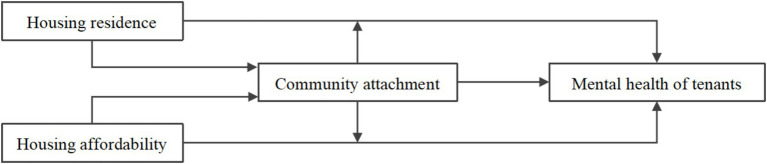
Theoretical framework of this study.

In summary, this study analyses the relationship between community attachment, housing quality, housing affordability and tenants’ mental health, thereby constructing a theoretical analysis framework (Figure 1). The proposed model conceptualizes housing quality and housing affordability as independent variables, while tenant mental health serves as the dependent variable. It further validates the role and mechanisms of community attachment within these relationships.

## 3 Data and methodology

### 3.1 Data collection

Through stratified sampling approach, one community was randomly selected from the villages that had been transformed into urban villages in each district of Shenzhen. After fully considering the feasibility of the survey, 7 communities of the research object were finally obtained.

**Figure 2:**
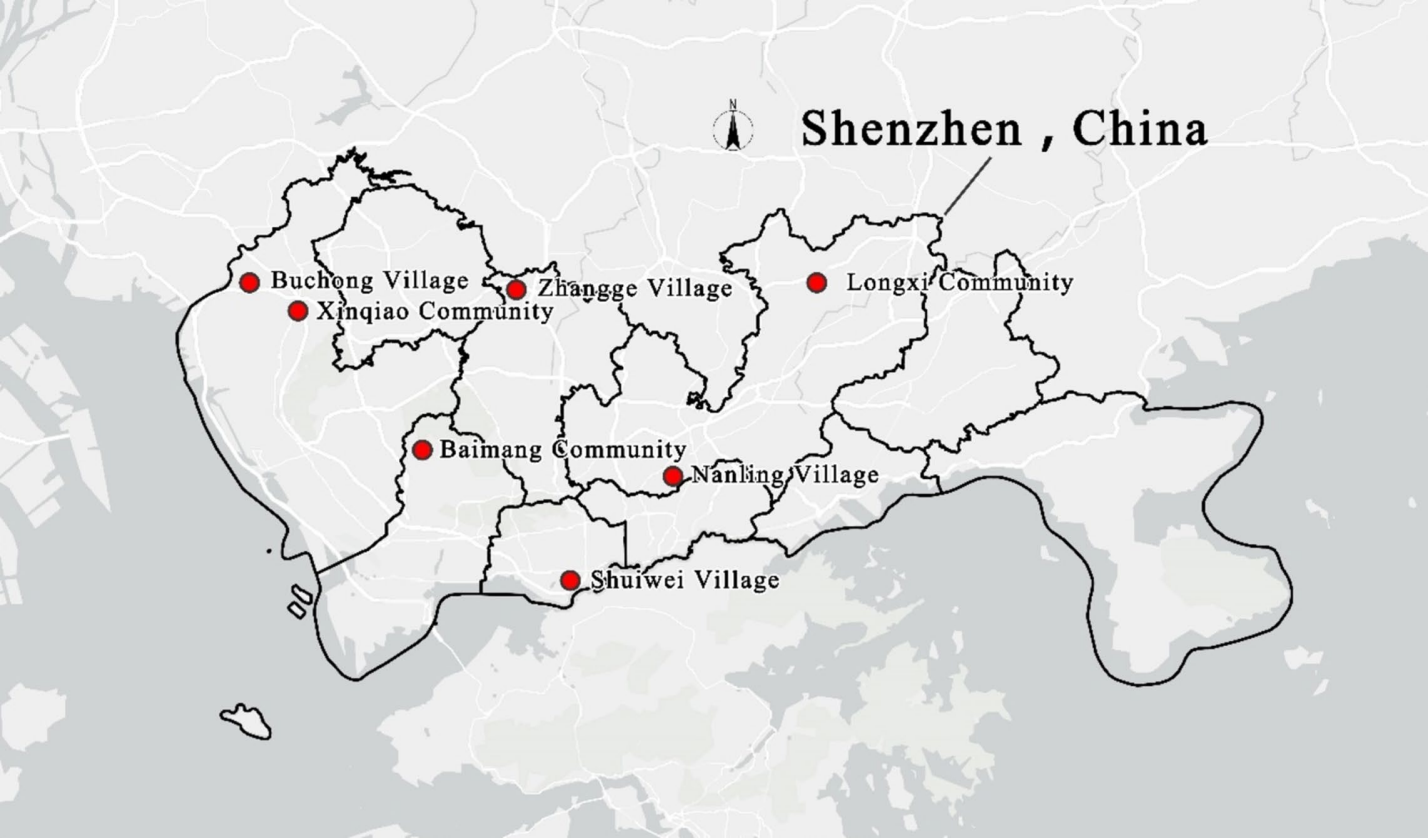
Map of the surveyed communities’ distribution.

The study used anonymous collection to distribute questionnaires from December 2023 to January 2024 to tenants within the following seven communities: the Nanling Village and Longxi Community in Longgang District, Shenzhen; the Buchong Village and Xinqiao Community in Bao’an District; the Zhangge Village in Longhua District; the Shuiwei Village in Futian District; and the Baimang Community in Nanshan District (Figure 2). Researchers coordinated with local community staff to facilitate questionnaires in the community during both working and non-working hours. A systematic sampling method was adopted to distribute questionnaires. Questionnaires were distributed every 10 households according to the house numbers of residents’ addresses until the target sample size was reached.

In this survey, a total of 750 questionnaires were distributed, of which 703 were deemed valid, yielding a valid response rate of 93.7. The questionnaire categorized the observed variables into five sections: housing quality in housing and community, housing affordability, community attachment, tenant mental health and individual economic and social attributes. The first four of these were obtained through subjective evaluations by the respondents, using a Likert scale with values ranging from 1 to 5 according to the degree of agreement or satisfaction.

### 3.2 Descriptive statistical analyses

This paper uses SPSS 26.0 software to statistically analyze the main characteristics of the individual economic and social attributes of the research samples, such as gender, age, marital status, and childbearing status (Table 1).

**Table 1:** Description of the samples’ socioeconomic characteristics.

Basic information	Classification	Count	Sample proportion	Average value
Gender	Male	378	53.8%	—
	Female	325	46.2%	—
Age		—	—	39
Marital status	Unmarried	140	19.9%	—
	Married	562	79.9%	—
	Else	1	0.1%	—
Fertility status	Zero child	159	22.6%	—
	One Child	210	29.9%	—
	Two children	281	40.0%	—
	Three children and above	53	7.5%	—
Academic qualification	Primary school	72	10.2%	—
	Junior high school	248	35.3%	—
	Senior high school	172	24.5%	—
	Junior college or undergraduate degree	206	29.3%	—
	Master’s degree or above	5	0.7%	—
Monthly income level	Less than 3,000 CNY	227	32.3%	—
	3,000–6,000 CNY	234	33.3%	—
	6,000–9,000 CNY	147	20.9%	—
	9,000–12,000CNY	47	6.7%	—
	12,000–15,000CNY	27	3.8%	—
	15,000 CNY or more	21	3.0%	—

The results showed that the gender distribution of the study samples was balanced with 53.8% male and 46.2% female. The average age of the respondents was 39 years. Regarding marital status, nearly two-thirds of the respondents were married.

Regarding fertility status 40.0% of respondents had two children—the most common category—while 22.6% had no children, 29.9% had one child, and 7.5% had three or more children. In terms of educational attainment, the proportions of respondents graduated from primary or secondary school, junior high school, senior high school, junior college or bachelor’s degree, and master’s degree or above were 10.2, 35.3, 24.5, 29.3, and 0.7%, respectively.

In terms of monthly income level, 32.3% of the respondents had a monthly income of “less than CNY 3,000,” 33.3% had a monthly income of “CNY 3,000-6,000,” 20.9% had a monthly income of “CNY 6,000-9,000,” and a smaller proportion had a monthly income of more than CNY 9,000.CNY.

Table 2 shows the descriptive statistics for each variable. The means of the respondents’ scores for housing quality, cost of residence, and community attachment all ranged from 3.182 to 3.707. The average rating score of respondents on the housing quality in urban villages was 3.71, reflecting that in general respondents rated urban villages relatively favorably. Respondents’ community attachment to the urban village was 3.56, also indicating that the surveyed tenants in general showed a potentially strong attachment to the urban village in which they live.

**Table 2:** Descriptive statistics of the means of the variables.

Variable name	N	Minimum value	Maximum values	Average value	Standard deviation
Housing quality	703	1.00	5.00	3.7073	0.69620
Community attachment	703	1.00	5.00	3.5602	0.72360
Housing affordability	703	1.00	5.00	3.1821	0.66097

Compared to the former two variables, surveyed tenants rated the cost of housing relatively low, with an average score of 3.18, reflecting the fact that housing affordability of living in a megacity urban village was perceived as relatively affordable.

In this study, the variable of mental health of tenants was calculated using the SF scale scoring method proposed about mental health (MH), with higher MH scores indicating better mental health. The results in Table 3 show the mental health scores of tenants in this survey and compared with the results of the quality-of-life research conducted by Wang et al. in five Chinese cities, the mental health scores of tenants in urban villages in Shenzhen were below the mean value of 77.61 (24).

**Table 3:** Scoring results of tenants’ mental health in the surveyed urban villages in Shenzhen.

Variable	Score ( $\bar{x}\pm s$ )	*t*-value	*p*-value
Mental health	74.6401 ± 12.6318	0.4909	0.001

### 3.3 Variable measurement

Housing quality: This study uses two dimensions (housing internal conditions and housing external conditions) of housing quality for evaluation (Table A-1). Among them, the evaluation of housing internal conditions includes indicators such as housing location, housing quality, housing spatial layout, and housing facilities. The evaluation of housing external conditions includes community public service facilities, community road, community environment and sanitation, community management and security.

Housing affordability: In the measurement of housing affordability (Table A-1), this study combines three aspects: living expenses, rental costs, and commuting costs.

Community attachment: The measurement of community attachment used in this study (Table A-1) is proposed by Williams et al.’s research, which measures six dimensions, including community meaning, community identity, community nostalgia, community pride, community significance and community belonging (25–27).

Mental health: Tenant mental health was the dependent variable in this study. This study used the medical outcomes study 36-item short form health survey (SF-36), which evaluates the dimensions of nervousness, dumps, calmness, mood, and happiness.

### 3.4 Tests of reliability and validity of the variables

In this study, the internal consistency of the variables was tested through the Cronbach′s a coefficient to ensure the data quality of the measurements and to ensure that the next analyses could be carried out. The results of the Cronbach′s a coefficient test for each dimension of the variables shows that the Cronbach coefficient values corresponding to the dimensions included in the variables of this study are all greater than 0.80, and the total correlations of the correction terms are all greater than 0.6, which indicates that the dimensions of the variables of the present study have high reliabilities, and all of them have good internal consistency.

To test the multicollinearity between the factors and to exclude the factors with small effects, for the validity test this study conduct exploratory factor analysis on the variables (Table 4). The principal component extraction method was used to extract the common factors and the extracted common factors were rotated using the variance maximization orthogonal rotation method, and the factor loading values of the measured indicators after orthogonal rotation ranged from 0.76–0.93, which indicates that the observed variables are affected by the latent variables with a high intensity, and all of them can be explained by the latent variables in a better way, and therefore the validity of the observed variables is reliable.

**Table 4:** Results of the exploratory factors analysis.

Dimension	Variable name	Factor load	Cronbach’s Alpha	Eigenvalue	Cumulative variance contribution(%)
Housing quality	Housing location	0.877	0.823	44.850	27.852
	Housing quality	0.866	—	—	—
	Housing spatial layout	0.841	—	—	—
	Housing Facilities	0.808	—	—	—
	Community public service facilities	0.821	—	—	—
	Community road	0.819	—	—	—
	Community environment and sanitation	0.844	—	—	—
	Community management and security	0.820	—	—	—
Housing affordability	Daily living expense	0.899	0.918	8.792	42.195
	Rental costs	0.900	—	—	—
	Commuting cost	0.902	—	—	—
Community attachment	Community meaning	0.894	0.902	15.662	65.255
	Community identity	0.898	—	—	—
	Community nostalgia	0.888	—	—	—
	Community pride	0.905	—	—	—
	Community significance	0.901	—	—	—
	Community belonging	0.902	—	—	—
Mental health of tenants	Nervousness	0.888	0.906	10.622	85.069
	Dumps	0.915	—	—	—
	Calmness	0.889	—	—	—
	Mood	0.904	—	—	—
	Happiness	0.921	—	—	—

### 3.5 Methods

This study used multiple linear regression models to examine the effects of housing quality and cost of residence on tenants’ mental health. Additionally, it analyzed the mediating and moderating roles of community attachment in the relationship between housing quality and tenants’ mental health.

The methodology used for the mediation effects test was mainly proposed by Baron and Kenny (28, 29). In addition, MacKinnon (30) proposed that the mediation effect can be tested by directly examining the “path coefficient of independent variable X to mediator variable M” and the “path coefficient of mediator variable M to dependent variable Y.” Therefore, this study uses the above method to validate the mediating effect in the proposed theoretical model.

Analysis of variance (ANOVA) was conducted when both the moderator and independent variables were categorical. If the interaction effect between the two is significant, the moderating variable has a moderating effect.

For the continuous moderator variable, hierarchical regression technique can be used to test the independent variables (31). Specifically, the size of the main effect of the independent variable and the moderator variable on the dependent variable is examined separately. Subsequently, the interaction term (independent variable × moderator variable) is included in the regression equation. A statistically significant coefficient for this item confirms the presence of a moderating effect.

## 4 Results

### 4.1 Impact of housing quality on tenants’ mental health

Multiple linear regression analyses were conducted with housing quality and housing affordability in urban villages as independent variables and tenants’ mental health as the dependent variable. It was found (Table 5) that the standardized coefficients of Models 2a and 2b improved in comparison to Model 1, indicating that both the housing quality and housing affordability have a significant positive effect on tenants’ mental health. Model 2c further compares the results of the models for the different independent variables, suggesting that housing quality has a slightly higher positive effect on tenants’ mental health than housing affordability.

**Table 5:** Results of multiple linear regression model analysis.

Variables	Model 1 (DV: mental health of tenants)			Model 2a (DV: mental health of tenants)			Model 2b (DV: mental health of tenants)			Model 2c (DV: mental health of tenants)		
	Coefficient	standard error	*p*	Coefficient	standard error	*p*	Coefficient	standard error	*p*	Coefficient	standard error	*p*
Housing quality	—	—	—	0.301^***^	0.045	0.000	—	—	—	0.228^***^	0.049	0.000
Housing affordability	—	—	—	—	—	—	0.295^***^	0.050	0.000	0.196^***^	0.054	0.000
Gender (reference group: male)	—	—	—	—	—	—	—	—	—	—	—	—
Female	−0.138^*^	0.076	0.069	−0.143^*^	0.073	0.051	−0.120*	0.074	0.104	−0.130^*^	0.073	0.074
Age	−0.009^**^	0.004	0.013	−0.010^**^	0.003	0.016	−0.008^**^	0.003	0.021	−0.009^**^	0.003	0.011
Academic qualifications (reference group: primary school)	—	—	—	—	—	—	—	—	—	—	—	—
Junior high school	−0.034	0.122	0.783	−0.055	0.118	0.643	−0.065	0.119	0.583	−0.071	0.117	0.547
Senior high school	−0.222	0.136	0.102	−0.223^*^	0.132	0.091	−0.232^*^	0.133	0.080	−0.230^*^	0.131	0.079
Junior college or undergraduate degree	−0.028	0.144	0.845	−0.051	0.139	0.714	−0.083	0.141	0.557	−0.082	0.139	0.555
Master’s degree or above	−0.220	0.404	0.585	−0.333	0.392	0.396	−0.521	0.397	0.190	−0.505	0.391	0.197
Marital status (reference group: unmarried)	—	—	—	—	—	—	—	—	—	—	—	—
Married	−0.127	0.215	0.555	−0.028	0.209	0.892	−0.137	0.210	0.513	−0.059	0.207	0.776
Else	0.639	0.495	0.124	0.655	0.475	0.145	0.620	0.480	0.151	0.612	0.468	0.164
Fertility status (reference group: zero child)	—	—	—	—	—	—	—	—	—	—	—	—
One child	−0.131	0.205	0.522	−0.162	0.199	0.415	−0.091	0.200	0.649	−0.128	0.197	0.516
Two children	−0.095	0.205	0.644	−0.120	0.199	0.547	−0.032	0.201	0.872	−0.073	0.198	0.713
Three children and above	0.288	0.235	0.222	0.243	0.228	0.287	0.328	0.230	0.153	0.281	0.227	0.216
Monthly income level	−0.032	0.032	0.323	−0.040	0.031	0.206	−0.049	0.032	0.127	−0.049	0.031	0.119
Constant	4.000^***^	0.210	0.000	2.890^***^	0.264	0.000	3.055^***^	0.259	0.000	2.531^***^	0.279	0.000
R^2^	0.061			0.117			0.107			0.134		

*, **, and *** indicate *p* < 0.10, *p* < 0.05, and *p* < 0.01, respectively.

### 4.2 Tests of the mediating effect of community attachment

The article combines two analytical methods, stepwise regression analysis and bootstrap, to test the mediating effect of community attachment (Tables 6**,** 7). With community attachment as the dependent variable in Model 3, the results show that housing quality and housing affordability all have significant positive effects on community attachment. In Model 4, we add the dependent variable of tenant’s mental health to Model 3, and the results show that community attachment has a significant positive effect in both housing quality-tenant’s mental health, and housing affordability-tenant’s mental health, and is an important factor influencing the relationship between the two.

**Table 6:** Results of the mediating effect of community attachment (1).

Variables	Model 3a (DV: Community attachment)			Model 3b (DV: Community attachment)			Model 4a (DV: Mental health of tenants)			Model 4b (DV: Mental health of tenants)		
	Coefficient	standard error	*p*	Coefficient	standard error	*p*	Coefficient	standard error	*p*	Coefficient	standard error	*p*
Housing quality	0.457^***^	0.036	0.000	—	—	—	0.115^**^	0.048	0.017	—	—	—
Housing affordability	—	—	—	0.445^***^	0.040	0.000	—	—	—	0.110^**^	0.051	0.031
Community attachment	—	—	—	—	—	—	0.406^***^	0.046	0.000	0.416^***^	0.045	0.000
Gender (reference group: male)	—	—	—	—	—	—	—	—	—	—	—	—
Female	0.026	0.057	0.657	0.060	0.059	0.307	−0.153^**^	0.070	0.028	−0.145^**^	0.070	0.038
Age	0.001	0.003	0.730	0.003	0.003	0.246	−0.010^***^	0.003	0.003	−0.009^***^	0.003	0.005
Academic qualifications (reference group: primary school)	—	—	—	—	—	—	—	—	—	—	—	—
Junior high school	0.032	0.092	0.731	0.016	0.095	0.863	−0.068	0.112	0.546	−0.072	0.112	0.521
Senior high school	−0.051	0.103	0.620	−0.065	0.106	0.537	−0.202	0.125	0.106	−0.205	0.125	0.102
Junior college or undergraduate degree	−0.078	0.109	0.474	−0.125	0.112	0.264	−0.019	0.132	0.883	−0.031	0.133	0.818
Master’s degree or above	−0.093	0.307	0.762	−0.374	0.316	0.237	−0.295	0.372	0.427	−0.366	0.375	0.331
Marital status (reference group: unmarried)	—	—	—	—	—	—	—	—	—	—	—	—
Married	−0.124	0.164	0.449	−0.290	0.167	0.083	0.022	0.199	0.912	−0.017	0.199	0.932
Else	0.661	0.484	0.334	0.613	0.491	0.382	0.687	0.431	0.174	0.665	0.432	0.179
Fertility status (reference group: zero child)	—	—	—	—	—	—	—	—	—	—	—	—
One child	0.058	0.156	0.711	0.166	0.159	0.299	−0.186	0.189	0.325	−0.160	0.189	0.398
Two children	−0.054	0.156	0.729	0.079	0.160	0.622	−0.098	0.189	0.603	−0.065	0.190	0.732
Three children and above	0.075	0.179	0.674	0.204	0.183	0.265	0.213	0.217	0.327	0.244	0.217	0.262
Monthly income level	0.012	0.025	0.622	−0.001	0.025	0.771	−0.045	0.030	0.135	−0.048	0.030	0.109
Constant	1.911^***^	0.206	0.000	2.177^***^	0.206	0.000	2.115^***^	0.265	0.000	2.150^***^	0.264	0.000
R^2^	0.206			0.177			0.215			0.205		

*, **, and *** indicate *p* < 0.10, *p* < 0.05, and *p* < 0.01, respectively.

**Table 7:** Results of mediating effect of community attachment (2).

Pathway	Effect type	Effect	LLCI	ULCI
Housing quality→Community attachment→Mental health of tenants	Total effect	0.3271^***^	0.2374	0.4169
	Direct effect	0.1317^***^	0.0364	0.2270
	Indirect effect	0.1954^***^	0.1379	0.2596
Housing affordability→Community attachment→Mental health of tenants	Total effect	0.3225^***^	0.2276	0.4175
	Direct effect	0.1397^***^	0.0423	0.2371
	Indirect effect	0.1828^***^	0.1286	0.2401

*, **, and *** indicate *p* < 0.10, *p* < 0.05, and *p* < 0.01, respectively.

Bootstrap analyses show that in the Path 1 model with housing quality as the independent variable, tenants’ mental health as the dependent variable, and community attachment as the mediator variable, the model indirect effect value was 0.195, with bootstrap confidence intervals ranging from 0.138 to 0.257, suggesting that the mediating effect of community attachment in the housing quality-tenants’ mental health relationship is relatively significant. In the path 2 model with housing affordability as the independent variable, tenants’ mental health as the dependent variable, and community attachment as the mediator variable, the model indirect effect value is 0.182, and the bootstrap confidence interval is 0.128–0.240, which suggests that community attachment has a significant mediating effect in the relationship between housing affordability and tenants’ mental health. Community attachment plays a more important mediating effect in these models.

### 4.3 Test of moderating effects of community attachment

In this study, two moderated effects tests were done using regression models with housing quality and housing affordability as independent variables, tenants’ mental health as dependent variable and community attachment as moderating variable, and the results of the tests are shown below (Tables 8**,** 9).

**Table 8:** Results of the moderating effects test for community attachment.

Variables	Model 5a			Model 5b			Model 5c		
	Coefficient	standard error	*p*	Coefficient	standard error	*p*	Coefficient	standard error	*p*
Housing quality	0.301^***^	0.045	0.000	0.115^**^	0.048	0.017	0.103^**^	0.047	0.028
Community attachment	—	—	—	0.406^***^	0.046	0.000	0.390^***^	0.045	0.000
Housing quality×Community attachment	—	—	—	—	—	—	0.292^***^	0.050	0.000
Gender (reference group: male)	—	—	—	—	—	—	—	—	—
Female	−0.143^*^	0.073	0.051	−0.153^**^	0.070	0.028	−0.142	0.068	0.105
Age	−0.010^***^	0.003	0.006	−0.010^***^	0.003	0.003	−0.009^***^	0.003	0.005
Academic qualifications (reference group: primary school)	—	—	—	—	—	—	—	—	—
Junior high school	−0.055	0.118	0.643	−0.068	0.112	0.546	−0.066	0.109	0.547
Senior high school	−0.223	0.132	0.191	−0.202	0.125	0.106	−0.206	0.122	0.189
Junior college or undergraduate degree	−0.051	0.139	0.714	−0.019	0.132	0.883	−0.034	0.129	0.790
Master’s degree or above	−0.333	0.392	0.396	−0.295	0.372	0.427	−0.518	0.365	0.157
Marital status (reference group: unmarried)	—	—	—	—	—	—	—	—	—
Married	−0.028	0.209	0.892	0.022	0.199	0.912	0.057	0.194	0.769
Else	0.755	0.475	0.145	0.587	0.431	0.174	0.475	0.414	0.187
Fertility status (reference group: zero child)	—	—	—	—	—	—	—	—	—
One child	−0.162	0.199	0.415	−0.186	0.189	0.325	−0.220	0.185	0.234
Two children	−0.120	0.199	0.547	−0.098	0.189	0.603	−0.139	0.185	0.452
Three children and above	0.243	0.228	0.287	0.213	0.217	0.327	0.110	0.212	0.605
Monthly income level	−0.040	0.031	0.206	−0.045	0.030	0.135	−0.047	0.029	0.105
R^2^	0.117			0.206			0.244		
Adjustment R^2^	0.101			0.190			0.228		
*F*-value	7.056			12.7733			14.788		
△R^2^	0.056			0.089			0.038		
△F	43.739			76.976			34.336		

*, **, and *** indicate *p* < 0.10, *p* < 0.05, and *p* < 0.01, respectively.

**Table 9:** Results of the moderating effects test of community attachment.

Variables	Model 6a			Model 6b			Model 6c		
	Coefficient	Standard error	*p*	Coefficient	Standard error	*p*	Coefficient	Standard error	*p*
Housing affordability	0.295^***^	0.050	0.000	0.110^**^	0.051	0.031	0.234^*^	0.057	0.096
Community attachment	—	—	—	0.416^***^	0.045	0.000	0.314^***^	0.045	0.000
Housing affordability×Community attachment	—	—	—	—	—	—	0.176^***^	0.059	0.003
Gender (reference group: male)	—	—	—	—	—	—	—	—	—
Female	−0.120	0.074	0.104	−0.145^**^	0.070	0.038	−0.145^**^	0.069	0.037
Age	−0.008^**^	0.003	0.021	−0.009^***^	0.003	0.005	−0.009^***^	0.003	0.006
Academic qualifications (reference group: primary school)	—	—	—	—	—	—	—	—	—
Junior high school	−0.065	0.119	0.583	−0.072	0.112	0.521	−0.072	0.112	0.520
Senior high school	−0.232^*^	0.133	0.080	−0.205	0.125	0.102	−0.219^*^	0.125	0.079
Junior college or undergraduate degree	−0.083	0.141	0.557	−0.031	0.133	0.818	−0.042	0.132	0.752
Master’s degree or above	−0.521	0.397	0.190	−0.366	0.375	0.331	−0.469	0.375	0.212
Marital status (reference group: unmarried)	—	—	—	—	—	—	—	—	—
Married	−0.137	0.210	0.513	−0.017	0.199	0.932	0.010	0.198	0.959
Else	0.720	0.480	0.051	0.465	0.432	0.079	0.309	0.429	0.115
Fertility status (reference group: zero child)	—	—	—	—	—	—	—	—	—
One child	−0.091	0.200	0.649	−0.160	0.189	0.398	−0.182	0.188	0.334
Two children	−0.032	0.201	0.872	−0.065	0.190	0.732	−0.096	0.189	0.610
Three children and above	0.328	0.230	0.153	0.244	0.217	0.262	0.194	0.217	0.371
Monthly income level	−0.049	0.032	0.127	−0.048	0.030	0.109	−0.044	0.030	0.142
R^2^	0.107			0.205			0.215		
Adjustment R^2^	0.090			0.189			0.198		
F-value	6.361			12.676			12.568		
△R^2^	0.046			0.098			0.010		
△F	36.266			84.722			8.985		

*, **, and *** indicate *p* < 0.10, *p* < 0.05, and *p* < 0.01, respectively.

Model 5a shows that housing quality exhibits significance at the 95% level (*p* < 0.05) on tenants’ mental health without considering the interference of community attachment as a moderating variable, suggesting that the housing quality have a significant effect on tenants’ mental health. Model 5b adds the moderating variable of community attachment, and Model 5c adds the variables of housing quality and community attachment to Model 5b. The results show that the interaction term of housing quality and community attachment exhibits significance at the 95% level (*p* < 0.05), indicating that there is a significant moderating effect of community attachment between housing quality and tenants’ mental health.

**Figure 3:**
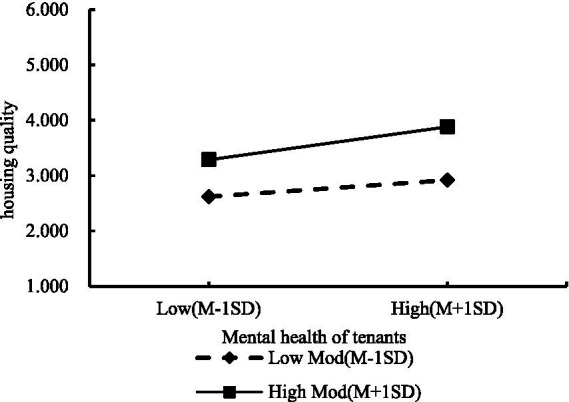
Slope plot of the moderating effect of community attachment on the relationship between housing quality and tenants’ mental health.

A slope plot based on the results of the moderating effects test (Figure 3) shows that different levels of community attachment moderate the relationship between housing quality and tenants’ mental health to varying degrees. At low levels of community attachment, housing quality plays a lesser role in positively influencing tenants’ mental health. The positive effect of housing quality on tenants’ mental health is greater at high levels of community attachment. Overall, it shows that community attachment has a positive moderating effect on the relationship between housing quality and tenants’ mental health.

In the moderating effects test with housing affordability as the independent variable, Model 6a shows that without considering community attachment as a moderating variable, Housing affordability exhibits significance at the 95% level on tenants’ mental health (*p* < 0.05), suggesting that housing affordability has a significant effect on tenants’ mental health. Model 2 adds the moderating variable of community attachment, and Model 6c adds the variables of housing affordability and community attachment to Model 6b, which shows that the interaction term of housing affordability and community attachment exhibits significance at the 95% level (*p* < 0.05), suggesting that community attachment has a significant moderating effect.

**Figure 4:**
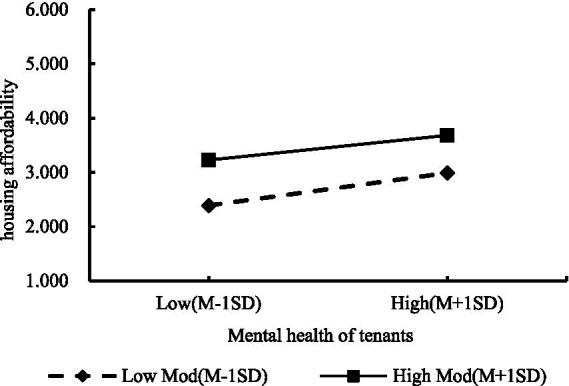
Slope plot of the moderating effect of community attachment on the relationship between housing affordability and tenants’ mental health.

Slope plots based on the results of the moderating effects test (Figure 4) indicates that different levels of community attachment moderate the relationship between housing affordability and tenants’ mental health to varying degrees. At low levels of community attachment, housing affordability plays a lesser role in positively influencing tenants’ mental health. At high levels of community attachment, housing affordability plays a larger role in positively influencing tenants’ mental health. Overall, it shows that community attachment enhances the relationship between housing affordability and tenants’ mental health.

## 5 Discussion and conclusion

This study analyses the relationship between housing quality, community attachment and mental health of urban village tenants, using seven urban villages in Shenzhen City as the research case. Housing quality and housing affordability are important factors affecting the mental health of tenants, and this study verifies the direct impact of housing quality ( β = 0.228, *p* = <0.05) and housing affordability ( β = 0.228, *p* = <0.05) on the mental health of tenants in urban villages. Therefore, effectively improving the housing quality and reducing the cost of residence can promote the mental health among urban village tenants.

The improvement in housing quality can be divided into two main components: the quality of housing and the living environment of the neighborhood. The quality of housing in existing urban villages mainly suffers from several problems, i.e., incomplete public service facilities, safety hazards, dilapidated buildings, lack of space for public activities, all of which have a long-term implication for the mental health of tenants.

Reducing the living cost in urban villages can also benefit the mental health of tenants to a certain extent. Housing affordability in this study is assessed through three components: daily living expenses (meals, clothing, etc.), rental costs, and commuting costs. In this survey, it is found that the residents’ evaluation of housing affordability was moderate, with a mean score of 3.18. However, some tenants perceived housing affordability as poor, likely due to the insufficient supply of affordable housing in the area of city center and the impact of recent urban village renewal policies in Shenzhen on rental prices. Thus, this suggests that the housing quality and housing affordability are interconnected factors in urban villages within China’s megacities.

In addition, this research tests the community attachment as a mediating and moderating variable, respectively. The finding indicated that community attachment has a mediating and moderating role in the relationship between housing quality and tenants’ mental health ( β = 0.292, *p* = <0.05), as well as housing affordability and tenant mental health ( β = 0.176, *p* = <0.05). Therefore, besides the direct effects of housing quality and housing affordability, fostering a stronger residents’ sense of community attachment can further enhance tenants’ mental health. Community attachment refers to the complex and comprehensive emotional bond that is formed between residents and their place, which has an imperceptible influence on the lives of residents. A positive sense of community attachment enables residents to experience greater happiness and fulfilment, thereby demonstrating more positive mental health.

Finally, this study proposes that the mental health of tenants should be an important consideration in the current urban village renewal in China’s megacities. Furthermore, the relationship between housing quality, housing affordability and community attachment should be dealt with in the urban village renewal projects.

Based on the above research, we also can find the contradictions in the process of urban village renewal in Shenzhen. While improving substandard housing conditions is essential for residents’ mental health, renewal efforts often lead to increasing housing prices, which may further affect affordability. Therefore, it is crucial to strike a balance between improving housing quality and maintaining affordable living costs. Shenzhen’s current urban village renewal policy adopts an inclusive approach, preserving the original rental market while encouraging state-owned enterprises to intervene in rental prices in areas with tight housing supply through unified purchase, renewal, and re-leasing of urban village properties. This strategy aims to maintain relatively low rental levels for residents, thereby enhancing living standards and protecting affordable rents. While this policy has alleviated some pressure on residents, the acquisition process remains complex. Ensuring the interests of both property owners and original tenants during acquisitions is a topic that warrants further investigation. Moreover, this research confirms that community attachment significantly influences residents’ mental health. Thus, promoting community cultural development and enhancing cohesion are essential topics in Shenzhen’s urban village renewal policies.

## Data Availability

The raw data supporting the conclusions of this article will be made available by the authors, without undue reservation.

## Appendix

**Table A1:** Description of the indicators measured.

Dimension	Variable name	Description	Standard for evaluation
Housing quality	Housing location	Location of housing	1 = very dissatisfied; 2 = dissatisfied; 3 = fair; 4 = satisfied; 5 = very satisfied
	Housing quality	Quality of construction and renovation of housing	1 = very dissatisfied; 2 = dissatisfied; 3 = fair; 4 = satisfied; 5 = very satisfied
	Housing layout	Spatial structure and layout within housing	1 = very dissatisfied; 2 = dissatisfied; 3 = fair; 4 = satisfied; 5 = very satisfied
	Housing Facilities	Housing facilities (lifts, utilities, gas, etc.)	1 = very dissatisfied; 2 = dissatisfied; 3 = fair; 4 = satisfied; 5 = very satisfied
	Community service facilities	Community public service facilities (hospitals, schools, activity areas for the older adult and children, etc.)	1 = very dissatisfied; 2 = dissatisfied; 3 = fair; 4 = satisfied; 5 = very satisfied
	Community road	Roads, traffic organization etc. within the community	1 = very dissatisfied; 2 = dissatisfied; 3 = fair; 4 = satisfied; 5 = vervy satisfied
	Community environmental sanitation	Green environment and hygiene of the community	1 = very dissatisfied; 2 = dissatisfied; 3 = fair; 4 = satisfied; 5 = very satisfied
	Community management and safety	Community management and community safety	1 = very dissatisfied; 2 = dissatisfied; 3 = fair; 4 = satisfied; 5 = very satisfied
Housing affordability	Daily living expense	I can afford daily living expenses (meals, clothing, etc.)	1 = very dissatisfied; 2 = dissatisfied; 3 = fair; 4 = satisfied; 5 = very satisfied
	Rental price	I can afford to rent a room	1 = very dissatisfied; 2 = dissatisfied; 3 = fair; 4 = satisfied; 5 = very satisfied
	Commuting cost	I can afford the commute	1 = very dissatisfied; 2 = dissatisfied; 3 = fair; 4 = satisfied; 5 = very satisfied
Community attachment	Community meaning	This community means a lot to me	1 = very dissatisfied; 2 = dissatisfied; 3 = fair; 4 = satisfied; 5 = very satisfied
	community identity	I think I belong in this community	1 = very dissatisfied; 2 = dissatisfied; 3 = fair; 4 = satisfied; 5 = very satisfied
	Community nostalgia	I would miss this neighbourhood if I moved away from it	1 = very dissatisfied; 2 = dissatisfied; 3 = fair; 4 = satisfied; 5 = very satisfied
	Community pride	I’m proud of the community I live in	1 = very dissatisfied; 2 = dissatisfied; 3 = fair; 4 = satisfied; 5 = very satisfied
	Importance of community	This community is very important to me	1 = very dissatisfied; 2 = dissatisfied; 3 = fair; 4 = satisfied; 5 = very satisfied
	Community belonging	This community gives me a sense of belonging	1 = very dissatisfied; 2 = dissatisfied; 3 = fair; 4 = satisfied; 5 = very satisfied
Mental health of tenants	Nervousness	Have you been a very nervous person?	1 = all of the time; 2 = most of the time; 3 = a good bit of the time; 4 = some of the time; 5 = a little of the time; 6 = none of the time
	Dumps	Have you felt so down in the dumps that nothing could cheer you up?	1 = all of the time; 2 = most of the time; 3 = a good bit of the time; 4 = some of the time; 5 = a little of the time; 6 = none of the time
	Calmness	Have you felt calm and peaceful?	1 = all of the time; 2 = most of the time; 3 = a good bit of the time; 4 = some of the time; 5 = a little of the time; 6 = none of the time
	Mood	Have you felt downhearted and blue?	1 = all of the time; 2 = most of the time; 3 = a good bit of the time; 4 = some of the time; 5 = a little of the time; 6 = none of the time
	Happiness	Have you been a happy person?	1 = all of the time; 2 = most of the time; 3 = a good bit of the time; 4 = some of the time; 5 = a little of the time; 6 = none of the time
